# Severe Photosensitivity Reaction After Pirfenidone Use

**DOI:** 10.7759/cureus.16626

**Published:** 2021-07-25

**Authors:** Saiara Choudhury, Pahnwat T Taweesedt, Rahul Dadhwal, Salim Surani

**Affiliations:** 1 Internal Medicine, Corpus Christi Medical Center, Corpus Christi, USA; 2 Pulmonary Medicine, Corpus Christi Medical Center, Corpus Christi, USA; 3 Anesthesiology, Mayo Clinic, Rochester, USA; 4 Medicine, Texas A&M University, College Station, USA; 5 Medicine, University of North Texas, Dallas, USA; 6 Internal Medicine, Pulmonary Associates, Corpus Christi, USA; 7 Clinical Medicine, University of Houston, Houston, USA

**Keywords:** pirferidone, idiopathic pulmonary fibrosis, photosensitivity reaction, ipf, dyspnea

## Abstract

Idiopathic pulmonary fibrosis is a chronic and progressive disease with a significant mortality rate. Pirfenidone is one of two oral antifibrotic therapies approved to treat idiopathic pulmonary fibrosis (IPF). Pirfenidone helps decrease disease progression in patients with IPF and reduces vital capacity. This has led to widespread use of this medication in recent years. In this case report, we present a 60-year-old male who started treatment with pirfenidone for IPF and had severe skin reactions after initiation of therapy.

## Introduction

Idiopathic pulmonary fibrosis (IPF) is a devastating and progressive fibrotic lung disease that is a form of interstitial lung disease. While the clinical course of the disease may vary, it has a median survival of only 2-5 years after diagnosis [1]. The most common presenting symptoms include cough, fatigue, and progressive shortness of breath [2]. It is common in males over 50 years old. Risk factors include a history of tobacco use, genetic predisposition, toxic occupational and environmental exposures, possible history of viral illnesses, and other comorbidities, like gastroesophageal reflux (GERD) [3]. The pathophysiology of IPF is thought to be due to injury to the lung parenchyma leading to inflammation and fibrotic changes [3]. Pirfenidone is an oral antifibrotic medication approved for treating patients with IPF in Japan and Europe in 2011, and it was subsequently approved in the United States in 2014 [1]. In 2015, according to the American Thoracic Society guidelines, pirfenidone was identified as one of two agents recommended for treatment of IPF, with the other one being nintedanib (Ofev®) [1]. The meta-analysis by Ren et al. showed that most reported side effects with pirfenidone use included gastrointestinal effects, like nausea and photosensitivity reaction [1,4]. While a few cases of adverse dermatologic reactions with pirfenidone use have been reported, the etiology of skin reactions using this medication is unclear [5].

## Case presentation

**Figure 1:**
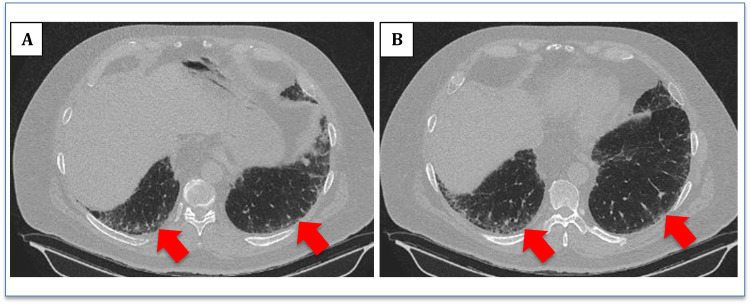
(A, B) CT scan of the chest (lung window) showing mild subpleural fibrosis (red arrows)

A 60-year-old male with a past medical history of obstructive sleep apnea and compliant with the nightly continuous positive airway pressure (CPAP) use, factor V Leiden deficiency with a history of deep vein thrombosis of the left lower extremity and pulmonary embolism 11 years ago (currently on oral anticoagulation), hypertension, hyperlipidemia, and obesity presented to the pulmonary outpatient clinic for nonproductive cough for six months and worsening shortness of breath on exertion. Computed tomography angiography (CTA) chest was done negative for acute pulmonary embolism. It showed mild to moderate interstitial pulmonary fibrosis in the lung bases showing the progression from a prior imaging study, consistent with early IPF (Figure 1).

**Figure 2:**
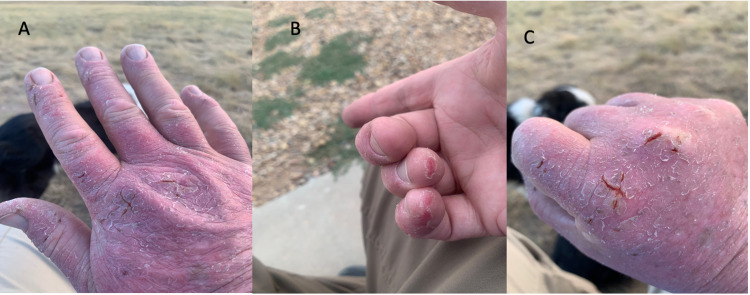
(A-C) Eczematous changes and skin breakdown consistent with photosensitive reaction due to exposure to sun

Pulmonary function test showed reduced lung volume, decreased lung capacity, and diffusion defect consistent with an interstitial process: forced vital capacity (FVC): 3.71 L (77% predicted), forced expiratory volume in one sec (FEV1): 2.9 L (78% predicted), FEV1/FVC: 80% total lung capacity (TLC): 5.27 L (72% predicted), diffusion capacity of carbon monoxide (DLCO): 20.84 mL/min/mm hg (61% predicted). The patient was started on oral pirfenidone at one tablet (267 mg/tablet) three times a day and increased to three tablets (267 mg/tablet) three times a day. Four months after starting therapy with pirfenidone, the patient reported developing a painful rash of both arms, hands, knees, all fingers, upper back, and shoulders. The patient reported using gloves and protective clothing when exposed to the sun without improvement in the rash. The images of the patient's rash are shown in Figure 2.

Dose reduction was attempted, but due to persistent painful rash, the pirfenidone was discontinued. Patient collagen vascular profile with rheumatoid arthritis (RA) factor and antinuclear antibody (ANA) was negative. The patient was given a course of oral steroids. Gradually over a month after discontinuation of pirfenidone, his skin findings resolved. He did not report any other medication change or exposure to other agents during this time. Thus, a diagnosis of photosensitivity reaction due to pirfenidone was made. The patient was re-evaluated in the pulmonary clinic for symptoms of dyspnea on exertion and started on therapy with nintedanib, which he tolerated well.

## Discussion

Pirfenidone (Esbriet®) was the first medication that was developed to treat IPF [5]. It is an oral antifibrotic and anti-inflammatory agent that has been shown to improve outcomes, specifically reduced decline in lung function, decreased progression of the disease, and reduced change in forced vital capacity in patients with IPF [1]. Lancaster et al. describe multiple studies in patients with IPF evaluating the long-term safety profile of this medication. While pirfenidone generally was found to have a favorable safety profile, multiple treatment-derived adverse effects were described as well. Most reported side effects included gastrointestinal symptoms (nausea, diarrhea), bronchitis, fatigue, and rash. Adverse skin effects were thought to be more common in the first six months of therapy. Dose reductions have also been reported to avoid these side effects [1]. It has been recommended to counsel patients on wearing sun-protective clothing and SPF to prevent photosensitivity reactions [1,6]. In our patient's case, his symptoms started a few months after the initiation of therapy. They persisted despite being on the lowest dose of the medications and taking precautions with sun exposure.

While there have been multiple reported cases of pirfenidone-induced phototoxic effect, the mechanism of phototoxicity remains unclear [6-8]. A study by Seto et al. discusses in vitro evidence of phototoxicity via generation of reactive oxygen species (ROS) via peroxidation of photodynamic lipids and cleavage of DNA due to exposure to sunlight [9]. The mainstay treatment of this photosensitivity reaction includes cessation of the drug and use of oral and topical steroids for symptom control. Since pirfenidone remains new in the market, it is essential to continue obtaining more data to get an exact incidence and prevalence of this phenomenon.

## Conclusions

Pirfenidone remains an essential drug for the treatment of IPF. However, it is vital to be cautious of side effects associated with its use, especially debilitating skin changes. Physicians prescribing this medication should counsel their patients of this possible side effect and encourage them to apply sunscreen and sun-protective clothing for prevention.
